# Eco-evolutionary dynamics in microbial interactions

**DOI:** 10.1038/s41598-023-36221-1

**Published:** 2023-06-03

**Authors:** Akihiko Mougi

**Affiliations:** Institute of Agricultural and Life Sciences, Academic Assembly, Shimane University, 1060 Nishikawatsu-Cho, Matsue, 690-8504 Japan

**Keywords:** Ecology, Evolution, Microbiology, Ecology

## Abstract

Microbes play an important role in ecosystem functioning and human health. A key feature of microbial interactions is a feedback system in which they modify the physical environment and react to it. Recently, it has been shown that the ecological consequences of microbial interactions driven by the modification of their surrounding pH environment can be predicted from the effects of their metabolic properties on pH. The optimum environmental pH for a given species can adaptively change in response to the changes in environmental pH that are induced by them. However, the mechanisms underlying the effect of these adaptive changes in pH niche on microbial coexistence are yet to be explored. In this study, I theoretically demonstrate that ecological theory can only accurately predict the qualitative ecological consequences if the growth and pH change rates are the same for each species, which suggests that adaptive pH niche changes can generally make ecological consequence predictions based on ecological theory difficult.

## Introduction

Vast amounts of diverse microbes coexist on Earth and play important roles in the ecological functioning of nearly all ecosystems^1–5^. In fact, microbes even form an ecosystem within the human body, with important effects on health^6–10^. Microbes interact with their environment in ways that alter their survival and growth and affect community composition^1–5^. A key feature in many microbial interactions is environmental modification^11–14^, which occurs when microbes modify their environment by consuming resources and excreting metabolites. Such environmental changes can affect the growth rate of coexisting microbes and results in a feedback loop that consists of changing the environment and reacting to it.

Environmental pH is a key factor for microbes^15–18^ due to its effects on protein and lipid membrane stability and the crucial role pH plays in the vital activities of microbes^19,20^. Microbes prefer pH values that optimize their growth rate^15,16^. Deviations from this ideal inhibits growth and promotes cell death^17,18^. Since microbes change their surrounding environmental pH values via their metabolic activities^18,21,22^, the existence of other bacterial species in their environment can inhibit or promote growth. A recent study showed that the ecological consequences of a microbial interaction driven by pH modification can be predicted from a combination of the effect of their metabolic properties on pH^23^. For example, when two bacterial species prefer very different pH environments (i.e., alkaline and acidic environments) and they both alter the pH in the same direction as their own pH preference, bistable coexistence occurs, although one species can be more abundant than the other depending on the initial environmental conditions^23,24^. In contrast, if they each alter the environmental pH in the opposite direction of their own pH preferences, then a globally stable coexistence occurs independent of the initial conditions^23^. This ecological theory is an important part of our understanding of the microbial system of environmental modification, but the question of how such interaction types or pH preferences evolve remains unanswered.

Experimental studies have shown that the pH preferences (i.e. pH niche) of microbes can adapt in response to a change in the environmental pH^25–29^ to maintain their pH homeostasis, even if this adaptation comes with a cost^30^. This suggests that bacterial species can adaptively change their pH niche in response to the changes in environmental pH caused by their own interactions with the environment. However, it remains unexplored how various microbial pH modifications affect their pH adaptation and ecological coexistence. Here, I consider the eco-evolutionary dynamics of two bacterial species that indirectly interact through the pH modifications they each cause in the environment and how each species alters their pH niche as a result (see “Methods”). One type of bacteria increases pH (alkaline-producing bacteria), whereas the other type decreases pH (acid-producing bacteria). Since pH is a key parameter that affects their growth rates, a pH that deviates from their preferred pH niche decreases their growth rates. Each bacterial species has a physiologically optimal pH. The adaptive change in pH niche can alter the optimal pH, which involves a cost (evolutionary constraint). Even with this constraint, microbes need to change the pH niche because a mismatch between environmental pH and pH niche can decrease the fitness. Feedback between ecological population dynamics and evolutionary dynamics may affect the relationship between the ecological consequences of microbial species interactions and their metabolic properties on pH. The eco-evolutionary dynamics model can have multiple equilibria. A key ecological consequence to consider is whether evolution results in a unique ecological equilibrium or multiple equilibria. Another consideration is whether the equilibrium is stable or not. The stability is characterized by resilience, which is the recovery rate back to equilibrium after a small perturbation, because the index is appropriate for representing the stability of each equilibrium in multiple equilibria and can be measured experimentally. The present study aims to show (i) how pH niches evolve through bacterial interactions and how this evolution determines various interaction types; and (ii) how evolutionary changes in pH niches affect the ecological consequences of coexisting bacterial species.

## Results

**Figure 1:**
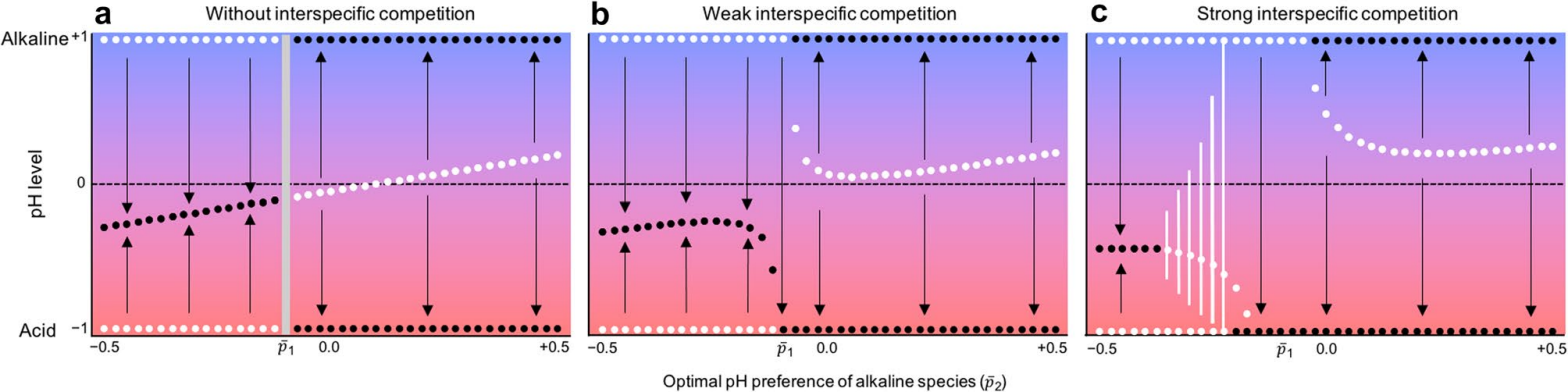
The major consequences of eco-evolutionary dynamics. In panels (**a–c**), the pH dynamics with various strengths of interspecific competition are illustrated. (**a**) Without interspecific competition (*α*_0*ij*_ = 0). (**b**) Weak interspecific competition (*α*_012_ = 0.1;* α*_021_ = 0.15). (**c**) Strong interspecific competition (*α*_012_ = 0.95;* α*_021_ = 1.4). Black and white points depict locally stable and unstable equilibria, respectively. The white lines in (**c**) represent the maximum and minimum values of pH oscillation. The arrows indicate the direction of pH change from a given initial pH value. The gray line in (**a**) denotes that the system is neutrally stable, with the dynamics determined by the initial conditions. Note that interspecific competition makes the neutral stability disappear and creates a uniquely stable acidophilic equilibrium. The equilibrium points and the local stability are numerically calculated. The stability is determined by the sign of a real part of the dominant eigenvalue of the Jacobian matrix [negative (positive) is stable (unstable)]. The amplitude of pH oscillation in (**c**) was calculated by a sufficient long-term simulation (t = 3 × 10^4^) where asymptotic behavior was obtained (initial values were: *X*_*i*_(0) = 0.1; *Y*(0) = 0; *p*_*i*_(0) = 0). The parameters were *k*_*i*_ = 0.1;* r*_*i*_ = 1.0; *G*_*i*_ = 0.01; *c* = 5; *θ* = 3; *δ* = 2; and ${\overline{p} }_{1}$= − 0.1.

**Figure 2:**
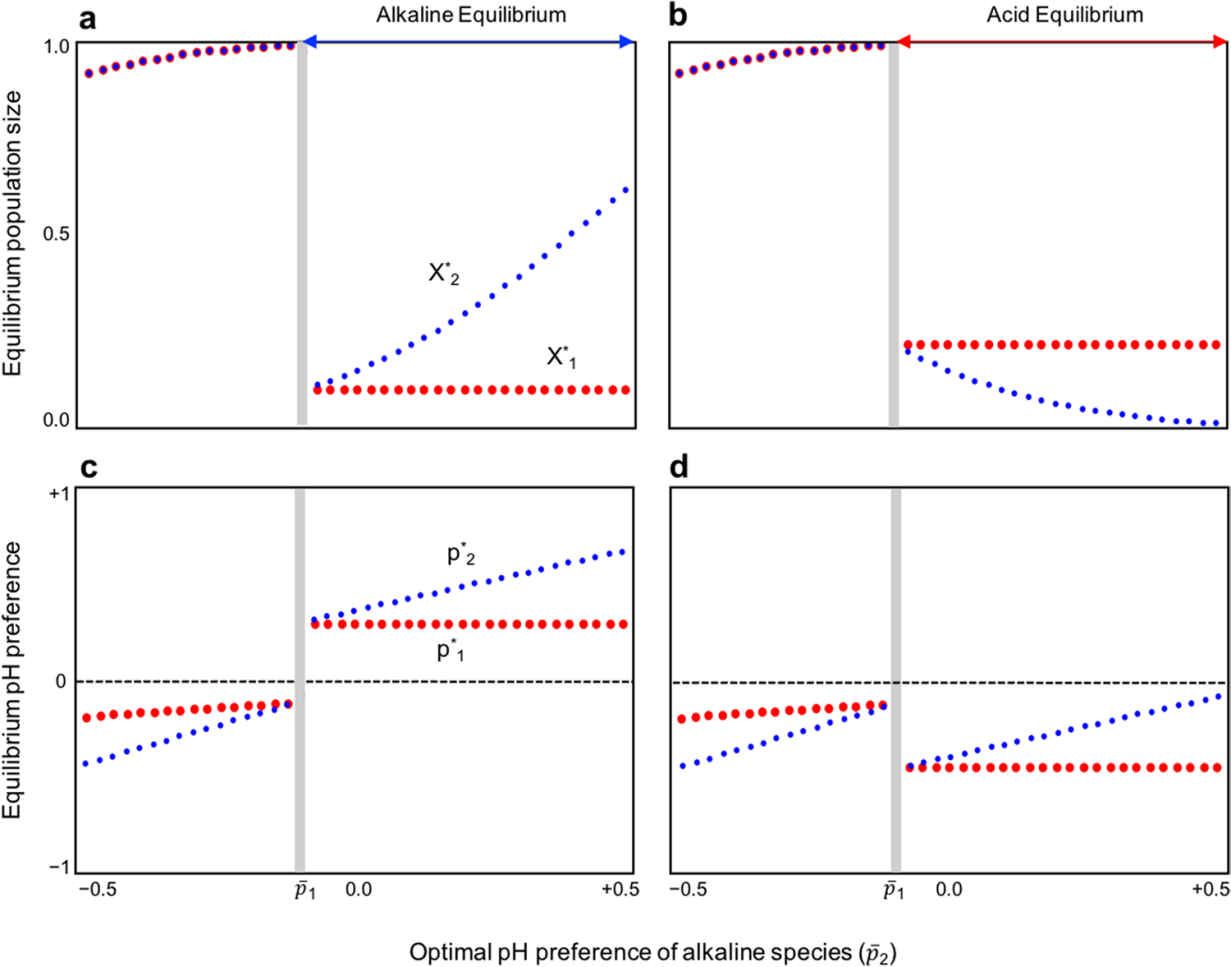
A typical pattern of equilibrium population size and trait values without interspecific competition. In (**a**,**b**) and (**c**,**d**), the equilibrium points of the population size and trait values are illustrated, respectively. In a bistable regime (${\overline{p} }_{2}>{\overline{p} }_{1}$), the system has different equilibria. (**a**,**c**) Alkaliphilic equilibrium. (**b**,**d**) Acidophilic equilibrium. Note that in the areas where ${\overline{p} }_{2}<{\overline{p} }_{1}$, the two cases have the same equilibrium. When ${\overline{p} }_{2}<{\overline{p} }_{1}$, then *X*_1_^*^ = *X*_2_^*^. The gray lines depict that the system is neutrally stable and the dynamics were determined by the initial conditions. The parameters were the same as those in Fig. 1a.

**Figure 3:**
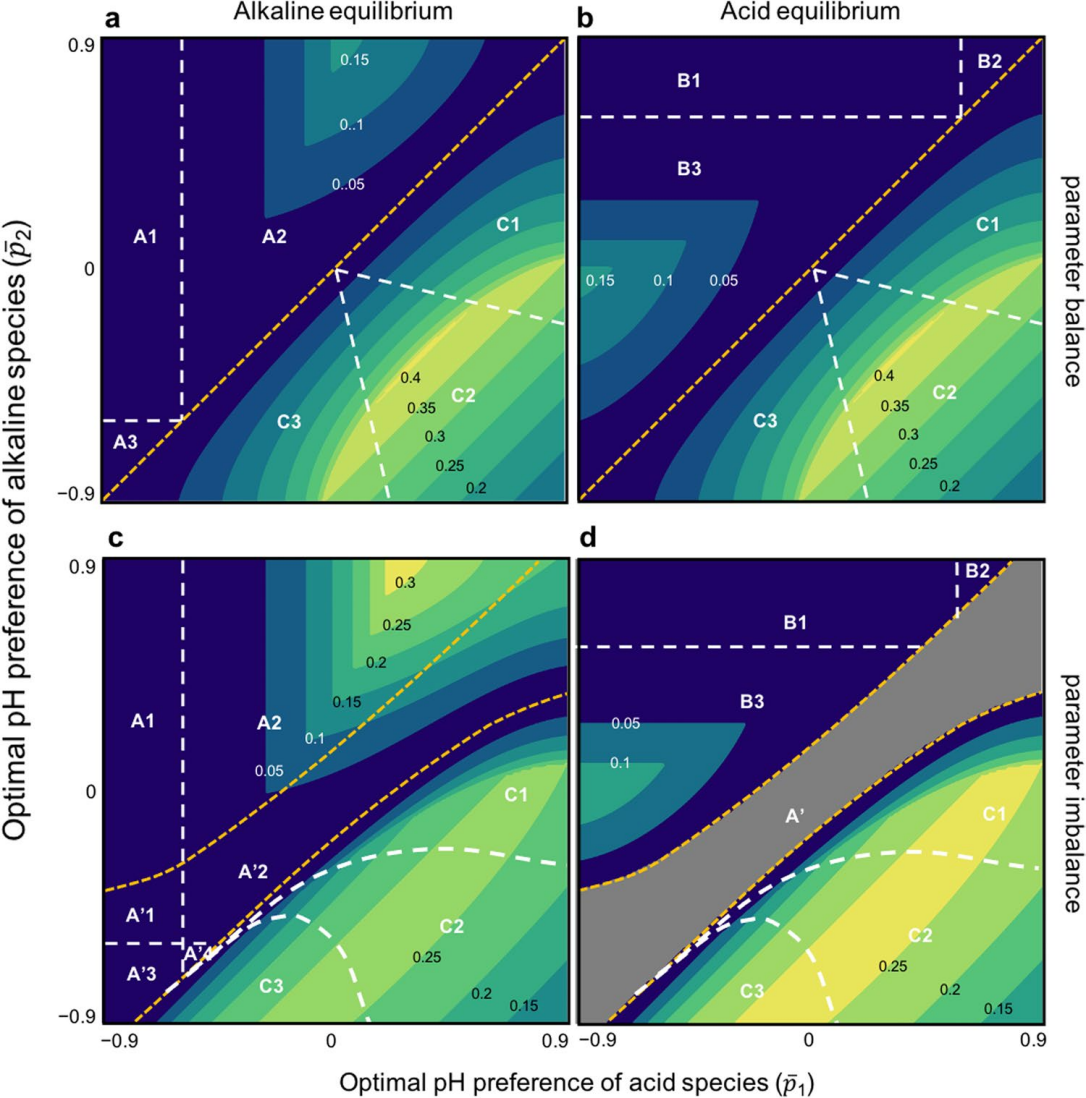
Resilience of equilibrium without interspecific competition. The resilience is illustrated in two cases: (**a**,**b**) Parameter balance (*k*_1_*r*_01_ = *k*_2_*r*_02_). (**c**,**d**) Parameter imbalance (*k*_1_*r*_01_ $$\ne$$ *k*_2_*r*_02_). In (**a**,**c**) and (**b**,**d**), the alkaliphilic and acidophilic equilibria are plotted, respectively. The contours represent the level of resilience (given by numbers). A, B and C indicate the different parameter spaces with different size relations of equilibrium trait values, in alkaliphilic, acidophilic and intermediate equilibrium, respectively. In (1–3) in A and B, (1) *p*_1_^*^ < 0 < *p*_2_^*^; (2) 0 < *p*_1_^*^ < *p*_2_^*^; and (3)* p*_1_^*^ < *p*_2_^*^ < 0. In (1–3) in C, (1) 0 < *p*_2_^*^ < *p*_1_^*^; (2) *p*_2_^*^ < 0 < *p*_1_^*^; and (3)* p*_2_^*^ < *p*_1_^*^ < 0. A′ indicates a region with a unique stable alkaliphilic equilibrium. In A′, the numbers correspond to the regions shown in (**a**), except for A′4, where *p*_2_^*^ < 0 < *p*_1_^*^. Note that in C, the intermediate equilibrium has the same resilience among (**a**) and (**b**) or (**c**) and (**d**). Orange dashed lines represent the thresholds separating the different regimes. In (**a**,**b**), the threshold is: $${\overline{p} }_{1}={\overline{p} }_{2}$$. The lower and higher orange dashed lines in (**c**,**d**) indicate $${\widehat{p}}_{2}$$ and $$\overset{\lower0.5em\hbox{$\smash{\scriptscriptstyle\smile}$}}{p} _{2}$$, respectively (see SI text for details of the notation). Parameters are the same as those in Fig. 1a except for *k*_2_ = 0.2 in (**c**,**d**).

In a case without interspecific competition (*α*_*ij*_ = 0, where *α*_*ij*_ is the competition coefficient), the system has two regimes: a stable or bistable equilibrium, depending on the parameter conditions. If the parameters affecting the production rate are perfectly balanced (*k*_1_*r*_01_ = *k*_2_*r*_02_), which means a trade-off exists between pH changing rate *k*_*i*_ and maximum growth rate *r*_0*i*_ (or perfect symmetry of each parameter among species), the two regimes are switched at $\overline{p}_{1} = \overline{p}_{2}$, where $\overline{p}_{i}$ is the physiologically optimal pH value in each species* i* (see SI text). When the optimal pH for acid-producing bacteria is higher than that for alkaline-producing bacteria ($\overline{p}_{1} > \overline{p}_{2}$), the system has a uniquely stable equilibrium. In this case, the pH approaches an intermediate level (Fig. 1a), which is given by the mean of the physiological optimal trait values ($Y^{*} = (\overline{p}_{1} + \overline{p}_{2}$)/2, where Y^*^ is the pH equilibrium) (see SI text), and the population sizes of both species are maintained at a high level (Fig. 2a,b). The ratio of equilibrium population sizes is determined only by the ratio of *r*_0*i*_ (i.e., species with a larger value of *r*_0*i*_ have a larger population size; see SI text), and is perfectly balanced. In this regime, the trait values at evolutionary equilibrium are determined by both species’ physiological optimal trait values (see SI text). In addition, the preferred pH at the equilibrium (*p*_i_^*^) for acid-producing bacteria is always higher than that for alkaline-producing bacteria (*p*_1_^*^ > *p*_2_^*^) and those traits are likely to converge to intermediate values (Fig. 2c,d, SI text). The analysis showed that each type of bacteria (alkaline producing and acid producing) will evolve to prefer the pH made by the other bacteria (*p*_1_^*^ > 0 > *p*_2_^*^) (Fig. 3, Supplementary Fig. S1, see SI text). That is, it is not possible for both bacteria types to evolve to prefer the pH created by their own products. In this example, resilience, which is the recovery rate back to equilibrium after a small perturbation, tends to be higher when each species evolves to prefer the pH made by the other species (*p*_1_^*^ > 0 > *p*_2_^*^) (Fig. 3).

In contrast, when the optimal pH for acid-producing bacteria is smaller than that for alkaline-producing bacteria ($\overline{p}_{1} < \overline{p}_{2}$), the system has two stable equilibria: acidophilic (Y^*^ = − 1) or alkaliphilic equilibrium (Y^*^ = 1), and the system converges to either extreme pH environment, depending on the initial conditions (Fig. 1a). An initial high (low) pH is likely to lead to the alkaliphilic (acidophilic) equilibrium. Similarly, if one species is initially more abundant than the other species, then an equilibrium will be reached in which the latter species is less abundant. When alkaliphilic or acidophilic equilibria are reached, then either the alkaline- or acid-producing bacteria will be more likely to have the higher population size, respectively (Fig. 2a,b). When the species have the same *r*_0*i*_ values, the original population size ratio is always maintained (SI text). The trait values at evolutionary equilibrium are determined by the species’ own physiologically optimal traits, independent of the other species (SI text), which is contrary to the intermediate equilibrium. In addition, *p*_1_^*^ < *p*_2_^*^ is always maintained (Fig. 2c,d, SI text). The analysis showed that each bacteria type will evolve to prefer the pH created by their self-produced products (*p*_1_^*^ < *p*_2_^*^) (a remarkable case is *p*_1_^*^ < 0 < *p*_2_^*^) (Fig. 3, SI text). That is, it is not possible that both bacteria types evolve to prefer the pH environment made by the other bacteria type, which is in total contrast to the intermediate equilibrium. In addition, the trait values for the alkaliphilic equilibrium are larger than those of the acidophilic equilibrium for each species (Fig. 2c,d, SI text). In both equilibria, *p*_i_^*^ > 0 or* p*_i_^*^ < 0 (while keeping *p*_1_^*^ < *p*_2_^*^) is possible for both species (Fig. 3, SI text), which implies that both species can simultaneously evolve to prefer either the alkaline or acidic environment. This consequence is likely to occur when cost (*c*) is low, pH sensitivity (*θ*) is high, or physiologically optimal pH levels match the pH made by each species’ own products ($\overline{p}_{1} < 0 < \overline{p}_{2}$) (SI text, Supplementary Figs. S2–S5). In this regime, the resilience of the equilibria is asymmetrical, that is, the stability of one equilibrium is low when the stability of the other equilibrium is high (Fig. 3). This asymmetry is remarkable when cost (*c*) or pH sensitivity (*θ*) is high (Fig. 3, Supplementary Figs. S2–S5). The resilience of both equilibria is low in a broad parameter range (Fig. 3).

Next, the perfect parameter balance was relaxed (*k*_1_*r*_01_ $\ne$ *k*_2_*r*_02_). This parameter imbalance had four major effects on the intermediate equilibrium. First, it created a third regime in which a uniquely stable equilibrium was reached with the system converging to one extreme pH environment (Supplementary Fig. S6). Accordingly, the parameter ranges of the intermediate equilibrium and the bistable equilibrium narrowed (Supplementary Fig. S6), which implies that the pH environment tends to be biased to one side. For example, when an alkaline-producing species is more productive than an acid-producing species (*k*_1_*r*_01_ < *k*_2_*r*_02_), the pH environment is likely to be alkaline (Supplementary Fig. S6). Second, the equilibrium population sizes (*X*_i_^*^) in the intermediate equilibrium became asymmetrical. The ratio of the population sizes was only determined by the ratio of pH change rates (*X*_1_^*^/*X*_2_^*^ = *k*_2_/*k*_1_) (SI text). Interestingly, the bacterial species with the higher pH change rate became inferior. In addition, due to the parameter imbalance, the population sizes in the intermediate equilibrium decreased as the physiologically optimal trait values are similar (Supplementary Fig. S6, SI text), which is in contrast to the perfect parameter balance scenario. Third, both species evolved to prefer the pH made by the bacterial species with the higher parameter rate (*k*_*i*_*r*_0*i*_*;* Supplementary Fig. S6, SI text). Fourth, it could increase or decrease resilience when *k*_*i*_ or *r*_*i*_ was asymmetrical between the species, respectively (Fig. 3c,d, Supplementary Fig. S7). The parameter imbalance had a smaller effect on the alkaliphilic and acidophilic equilibria than it did on the intermediate equilibrium state. The only exception was resilience, which was increased by the parameter imbalance (Fig. 3c,d, Supplementary Fig. S7).

The parameter imbalance has another crucial effect on the qualitative prediction of ecological consequences resulting from the model with or without evolution. First, in a case with perfect parameter balance, thresholds separating different regimes in the systems with or without evolution are exactly the same (SI text). In contrast, parameter imbalance alters these thresholds. In a scenario without evolution (a very large *c*), thresholds separating different regimes do not depend on the cost *c*, contrary to the system with evolution. Hence, the qualitative prediction based on the physiologically optimal trait values can substantially differ among models with or without evolution, particularly when *c* is low (Supplementary Fig. S8a). A more appropriate comparison can be made when the trait values at evolutionary equilibrium are fixed in compatibility with the optimal level of each species. Even in this case, the qualitative predictions can differ (Supplementary Fig. S8b). For example, bistability is predicted in the model without evolution, while the third regime (monostable equilibrium with an extreme pH) is predicted in the model with evolution (Supplementary Fig. S8b).

Finally, consider interspecific competition (*α*_*ij*_ > 0). When competition is weak, the described results were not qualitatively different (Fig. 1b, Supplementary Figs. S9, S10). A uniquely stable acidophilic equilibrium arose within the narrow range between the intermediate and bistable equilibria regimes, because the inherent competitive ability is stronger in acid-producing bacteria. However, stronger competition (Fig. 1c) had five major effects. First, it widened the unique acidophilic equilibrium regime. Second, coexistence became impossible in the bistable regime (Supplementary Figs. S10, S11, S12). That is, one species was excluded depending on the initial conditions. Third, it widened the niche differences (SI text). Fourth, a limit cycle could occur in the intermediate equilibrium (Fig. 1c, Supplementary Figs. S10, S11, S12). Hence, pH could cyclically change and bacteria could coexist in oscillation (Supplementary Fig. S13). This cycle is likely to occur when the cost of changing trait (*c*) is large, pH sensitivity (*θ*) is high, and niche width (*σ*) is wide (Supplementary Fig. S14). Fifth, the speed of adaptation (*G*_*i*_) can largely affect the dynamics. Slower adaptation is likely to stabilize the oscillating population levels (Supplementary Fig. S15). The faster adaptation of the inferior competitor did not prevent stabilization, but the faster adaptation of both species was more likely to destabilize the population dynamics (Supplementary Fig. S15).

## Discussion

The present study demonstrates that in a microbial system where two bacterial species interact through the pH modifications they create in the environment, the evolutionary dynamics of each species’ preferred pH levels greatly affect the ecological population dynamics and coexistence of the bacteria. The eco-evolutionary dynamics can lead to major consequences. In simple cases with a perfect balance between production parameters, when $\overline{p}_{1} < \overline{p}_{2}$, both species tend to change the existing environmental pH to the one favorable to itself, causing positive feedback and bistability in which pH niches of both species evolve to adapt to either alkaline or acidic environments depending on an initial condition. On the other hand, when $\overline{p}_{1} > \overline{p}_{2}$, both species tend to change the existing environmental pH favorable to the competitor, leading to negative feedback and monostability in which pH niches of both species tend to evolve to adapt to an environment with near neutral pH. In cases with imperfect balance between production parameters, pH of the environment is likely to be more influenced by the highly productive species, causing a monostability in which the pH is changed to either extremes. Moreover, based on the present theory, eco-evolutionary dynamics can lead to qualitatively different predictions on ecological consequences resulting from those made based on ecological theory, particularly with parameter imbalance.

A recent study showed that the ecological consequences of two species that change and are affected by pH can be predicted by how each species affects pH^23,24^. The direction of pH change (i.e., an increase or decrease) and the pH preference (i.e., alkaliphilic or acidophilic) of each species can generally predict the ways in which they can coexist or go extinct, such as competitive exclusion and mutual extinction. The present study suggests that such predictions can differ over time as pH preferences evolve. If we predict ecological consequences without considering evolution, then we should see a relationship between the optimal pH preference of each species and the ecological consequences. If parameters (pH change rates and growth rates) are perfectly balanced, evolution does not affect the prediction of qualitative ecological consequences. However, disturbing the symmetry in parameters between two species can make the prediction of ecological consequences based on ecological theory difficult. The parameter imbalance creates a parameter region where the ecological consequences predicted from the two theories are qualitatively different. The same trait values in evolutionary stable state and physiologically optimum state can have different predictions of ecological consequences. This suggests that even if the two species in different systems have similar pH preferences, the two systems can show qualitatively different ecological consequences. While setting up an experimental system, it is crucial to determine whether the coexisting species used has a shared evolutionary history or not, which may critically influence the ecological consequences. In the absence of direct resource competition, since trait values at evolutionary equilibrium are not affected by the other species in alkaliphilic or acidophilic equilibrium, we need to predict the ecological consequences from the trait relationship at evolutionary equilibrium. In addition, in bistable equilibria, each species can have very different trait values, which implies that different trait relationships can predict similar ecological consequences. On the other hand, if two bacterial species change the pH in the same direction, then adaptive pH niche evolution halts the self-extinction of both species (see SI text). In addition, pH niche may not be steady state but rather cyclical, which can also increase prediction complexity.

Whether the pH preference at evolutionary equilibrium is affected by another species or not depends on the regime. This difference occurs regardless of whether the evolutionarily stable pH preferences of the two species are not far apart from their own physiologically optimal trait values. In the bistable regime, the equilibrium trait value is not far from the optimal value of both species. In an extreme case, each species prefers the pH environment made by their own products^23,24^, which results in each species hindering the growth of the opponent species. In such an antagonistic interaction, the bacterial species should simply prefer the pH environment made by their own products, regardless of the other species if resource competition is weak. In an intermediate equilibrium regime, the equilibrium trait value is far from the optimal value of both species. In an extreme case, each species prefers the pH environment made by the opponent’s products and consequently aids the survival of the opponent species^23,24^. In such a mutualistic interaction, each species should change their pH preferences to help the other species. For example, if one species prefers an extreme pH environment, then the other species should change their pH preference to adapt to the pH environment preferred by the opponent species. Consider a case where a species producing an alkaline product (alkaline-producing species) prefers a mildly acidic environment and the other species that produces an acidic substance prefers a strong alkaline environment (acid-producing species). The alkaline-producing species should change the pH environment to become more alkaline for the acid-producing species and evolve to prefer a higher pH level; however, the acid-producing species should not create a more acidic pH environment for the alkaline-producing species, because the alkaline-producing species is now adapted to the weaker acidic environment. These examples show that the presence of antagonistic or mutualistic interactions via pH makes a large difference in the eco-evolutionary dynamics of the system. Mutualistic interactions are expected to cause a gradual coevolution of pH preferences and to be stably maintained, whereas antagonistic interactions are expected to cause an intermittent evolution or evolutionary regime shift and to be fragile in the sense that the equilibrium states can change due to environmental fluctuations. More specifically, the ease of switching between equilibria depends on the direction that evolution takes.

Many organisms change the physical and chemical properties of the environment and react to environmental changes^31–33^. Plants change the soil, plankton change the light environment, and microbes change oxygen and metabolite concentrations. In plant–soil and ecosystem engineering systems^34–38^, a negative feedback stabilizing mechanism, such as in the mutualistic interaction in the present model, is known to be a key factor for competitive ecological coexistence. For example, a modification of the habitat by inhibiting the invasion of conspecific species causes an intraspecies negative feedback, allowing multiple competing species to coexist^38^. Competing plants can also coexist through a negative feedback caused by soil changes in favor of another species or a feedback that acts against own species^36^. These organism–abiotic environment feedback systems including the present study have in common the ability of self-regulation and helping other species, which can cause stable competitive coexistence. However, in diverse contexts, it remains unclear whether ecological coexistence is maintained on an evolutionary time scale. A key point for future studies is to reveal a general prediction of eco-evolutionary consequences in various systems of interactions between organisms and the abiotic environment.

## Methods

### Model formulation

Consider two types of bacteria that indirectly interact through the pH changes caused by each. One type increases pH (alkaline-producing bacteria), whereas the other type decreases pH (acid-producing bacteria). Since pH is a key parameter that affects their growth rates, a pH that deviates from their preferred pH niche decreases their growth rates. They also compete if they share a common niche. Here, the niche is assumed to be strongly related to their preferred pH environment, which implies that as their preferred pH becomes similar, the preferred habitats become similar and they are more likely to share common resources. The dynamics of bacterial population sizes and pH levels in this scenario are described by the following differential equations:

1a $$dX_{i} /dt = \, (r_{i} \left( {Y,p_{i} } \right) \, {-}X_{i} - \alpha_{ij} \left( {p_{i} ,p_{j} } \right)X_{j} )X_{i} ,$$

1b $$dY/dt = \, (k_{{2}} X_{{2}} {-}k_{{1}} X_{{1}} )({1}{-}Y^{{2}} ),$$

where *i* represents the type of bacteria (*i* ∈ 1, 2); *X*_1_ and *X*_2_ are the population size of acid-producing and alkaline-producing bacteria, respectively; *Y* is the pH level; *r*_*i*_ is the per capita growth rate; *α*_*ij*_ is the competition coefficient defined as the relative strength of interspecific competition to intraspecific competition; and *k*_*i*_ (> 0) is the rate of pH change caused by each bacteria. Here, pH is assumed to be self-regulated to avoiding divergence and pH can be in equilibrium as *Y* = 1 (alkaliphilic equilibrium) or *Y* = − 1 (acidophilic equilibrium). In addition, when *k*_2_*X*_2_ = *k*_1_*X*_1_, it can be in equilibrium with an intermediate pH level.

The growth rate of each bacteria is maximized at a preferred pH level, *p*_*i*_. If the preferred pH is a physiologically optimal value $\overline{p}_{i}$ (*p*_*i*_ $= \overline{p}_{i}$), the growth rate is described by an inverse bell-shaped function (*r*_*i*_ = ${r}_{0i}{e}^{{-\theta \left(Y-{p}_{i}\right)}^{2}}$) where *r*_0*i*_ is the maximum growth rate for each bacterial species and *θ* (> 0) is the shape parameter of the function (pH sensitivity). As *θ* increases, the steepness of the function increases, implying that pH preference is narrow. The pH preference is clearly crucial to bacterial fitness, which strongly suggests that pH preference is a key selection target trait that will evolve in response to changes in environmental pH.

To consider the evolution of pH preference in bacteria, the cost constraint for changing pH preference is assumed. Since a deviation from the physiologically optimal trait value is assumed to decrease the growth rate, the growth rate with a specific cost function is given by *r*_*i*_ = *r*_0*i*_ $e^{{ - \theta \left( {Y - p_{i} } \right)^{2} }} e^{{ - c\left( {\overline{p}_{i} - p_{i} } \right)^{2} }}$, where *c* represents cost strength. A large value of *c* implies that the trait change is very costly. In this study, pH is a key niche and an overlap of pH preferences among bacterial species implies an overlap of spatial and/or food resources. Thus, interspecific competition increases as the pH preferences of each bacterial species become more similar. A specific competition function is given by: *α*_*ij*_ = *α*_0*ij*_ ${e}^{{-\delta \left({p}_{i}-{p}_{j}\right)}^{2}}$, where *α*_0*ij*_ is the maximum competition coefficient in each bacterial species (the effect of species *j* on *i*) and *δ* (> 0) is the shape parameter of the function that reflects the niche breadth. A large value of *δ* implies that the niche breadth is very narrow, whereas a slight deviation between their trait values can greatly weaken interspecific competition.

The adaptive dynamics of the mean trait value (*p*_*i*_) are modeled by a quantitative trait evolution model^36^:

2 $$\frac{d{p}_{i}}{dt}={G}_{i}{\left.\frac{\partial {W}_{i}}{\partial {p}_{\mathrm{m}i}}\right|}_{{p}_{\mathrm{m}i}={p}_{i}},$$

where *G*_*i*_ (*i* $\in$ 1, 2) represents the control parameter of speed of adaptation^39^. Small values of *G*_*i*_ (< 1) can represent evolutionary processes that are slower than population dynamics, whereas larger values (> 1) can represent phenotypic plasticity that is faster than population dynamics. *W*_*i*_ represents the fitness of the mutant trait (*p*_m*i*_), which is defined as the per capita growth rate of the mutants: *W*_*i*_ = ${r}_{i}\left({p}_{\mathrm{m}i}\right)-{X}_{i}- {\alpha }_{ij}\left({p}_{\mathrm{m}i},{p}_{j}\right){X}_{j}$. Equation (2) indicates that the rate of adaptive change in the traits should be proportional to the selection gradient (${\left.\frac{\partial {W}_{i}}{\partial {p}_{\mathrm{m}i}}\right|}_{{p}_{\mathrm{m}i}={p}_{i}}$). If the selection gradient is positive, then selection pushes the population toward higher trait values, but if it is negative, then selection pushes the population toward lower trait values. At evolutionary equilibrium, the selection gradient is zero. Although there is a possibility that physiologically optimal trait value may evolve, it will evolve only on a longer time scale than the pH preference. For example, if a specifically similar pH environment continues over a very long time, the optimal trait value may evolutionarily change according to the pH value of the environment. On the other hand, environmental pH can also fluctuate in the short term. The evolution of preference for pH described in this study is expected to occur in a shorter time scale than the evolution of the optimal trait value. Hence, the present model considers a short time scale in which there is no change in the physiologically optimal trait value.

### Analytical procedure

Considering a simple case without direct interspecific competition for analytical tractability, the equilibrium between two coexisting species and the local stability of the equilibrium can be analyzed as follows.

The equilibrium (*X*_i_^*^, Y^*^,* p*_i_^*^) is obtained by considering that the differential Eqs. (1a, 1b, 2) are equal to zero. There are three pH equilibria: acidophilic equilibrium (Y^*^ = − 1), alkaliphilic equilibrium (Y^*^ = 1), and intermediate equilibrium (Y^*^ = $\frac{{\left( {1/c + 1/\theta } \right)\gamma }}{{2\left( {\overline{p}_{2} - \overline{p}_{1} } \right)}} + \frac{{\overline{p}_{1} + \overline{p}_{2} }}{2}$). In the extreme pH equilibrium, population equilibrium is calculated using the formula *X*_i_^*^ = *r*_0*i*_*F*_*i*_, where $F_{i} = e^{{ - \frac{{\left( {1 - \overline{p}_{i} } \right)^{2} }}{{\left( {1/c + 1/\theta } \right)}}}}$ denotes the alkaliphilic equilibrium and $F_{i} = e^{{ - \frac{{\left( {1 + \overline{p}_{i} } \right)^{2} }}{{\left( {1/c + 1/\theta } \right)}}}}$ denotes the acidophilic equilibrium. In the intermediate equilibrium, *X*_i_^*^ = *r*_0*i*_*E*_*i*_, where $E_{1} = e^{{ - \frac{{\left[ {\gamma \theta + c\left\{ {\gamma + \theta \left( {\overline{p}_{1} - \overline{p}_{2} } \right)^{2} } \right\}} \right]^{2} }}{{4c\theta \left( {c + \theta } \right)\left( {\overline{p}_{1} - \overline{p}_{2} } \right)^{2} }}}}$ and $E_{2} = e^{{ - \frac{{\left[ {\gamma \theta + c\left\{ {\gamma - \theta \left( {\overline{p}_{1} - \overline{p}_{2} } \right)^{2} } \right\}} \right]^{2} }}{{4c\theta \left( {c + \theta } \right)\left( {\overline{p}_{1} - \overline{p}_{2} } \right)^{2} }}}}$. The trait values in each pH equilibrium are given by *p*_Li_^*^ = $\frac{{c\overline{p}_{i} + \theta }}{c + \theta }$ (alkaliphilic equilibrium), *p*_Ci_^*^ = $\frac{{c\overline{p}_{i} - \theta }}{c + \theta }$ (acidophilic equilibrium), and *p*_Ii_^*^ = $\frac{{c\overline{p}_{i} }}{c + \theta } + \frac{ \theta }{{c + \theta }} \cdot \frac{{ \left( {\overline{p}_{1} + \overline{p}_{2} } \right)}}{2} + \frac{ \gamma }{{2c\left( {\overline{p}_{2} - \overline{p}_{1} } \right)}}$ (intermediate equilibrium).

I conduct a local stability analysis of the equilibrium, under a condition that the trait dynamics are very fast and at a quasi-equilibrium (SI text). Then, I have the stability criteria in alkaliphilic and acidophilic equilibrium: $$\overline{p}_{2} > \hat{p}_{2} = 1 - \sqrt {\left( {1 - \overline{p}_{1} } \right)^{2} - \gamma \left( {\frac{1}{c} + \frac{1}{\theta }} \right)}$$ and $$\overline{p}_{2} > \overset{\lower0.5em\hbox{$\smash{\scriptscriptstyle\smile}$}}{p}_{2} = - 1 + \sqrt {\left( {1 + \overline{p}_{1} } \right)^{2} - \gamma \left( {\frac{1}{c} + \frac{1}{\theta }} \right)}$$, respectively, where $$\hat{p}_{2}$$ and $$\overset{\lower0.5em\hbox{$\smash{\scriptscriptstyle\smile}$}}{p}_{2}$$ are the threshold of $$\overline{p}_{2}$$ in each equilibrium. When *γ* = 0, $$\hat{p}_{2} \, = \,\overset{\lower0.5em\hbox{$\smash{\scriptscriptstyle\smile}$}}{p}_{2} \, = \,\overline{p}_{1}$$.

## Supplementary Information

Supplementary Information.

## Data Availability

All data generated and analyzed during this study are included in this published article.
