# Supplementary material for: Eco-evolutionary dynamics in microbial interactions
Source: Sci Rep. 2023 Jun 3;13:9042. doi: 10.1038/s41598-023-36221-1 (PMC10239498; doi:10.1038/s41598-023-36221-1)

**Supplementary Information**

Eco-evolutionary dynamics in microbial interactions

**Akihiko Mougi**

**SI text (Appendix):**

At evolutionary equilibrium, the selection gradient in Eq. 2 in the main text becomes zero and the following condition must be met in each species:

$\frac{\delta F_{i}}{c+\theta}\left( p_{j}-p_{i} \right)=\frac{c\bar{p}_{i}}{c+\theta}+\frac{\theta Y}{c+\theta}-p_{i}$, (A1)

where *F_i_ = α_ij_X_j_*/*r_i_* represents the competitive effect of a second species that is normalized by the growth rate of the focal species. Then *δ*/(*c* + *θ*) in l.h.s. represents the relative strength of the competitive niche effect on the pH effect. The first and second terms in r.h.s. are the physiological optimal trait value and pH weighted by the relative cost and pH sensitivity effects, respectively. The equation A1 suggests that at evolutionary equilbrium, the niche difference in two competitive species must balance the deviation from the optimal trait value detemined by cost and pH envirnment. When both species are at evolutionary equilbrium, the diffrence in the traits of two species is:

$p_{i}^{*}{-p}_{j}^{*}=\frac{c\left( \bar{p}_{i}-\bar{p}_{j} \right)}{c+\theta-\left( F_{1}+F_{2} \right)\delta}$, (A2)

where the asterisks represent equilbrium. Note that the denominator in Eq. A2 is positive because $\frac{p_{i}^{*}{-p}_{j}^{*}}{\bar{p}_{i}-\bar{p}_{j}}>\frac{c}{c+\theta}$ , which is derived from the condition *F*_1_ + *F*_2_ > 0 at evolutionary equilibrium. Given this constraint, Eq. A2 indicates that (i) with higher cost or lower pH sensitivity, the difference in the traits at evolutionary equilbrium is almost entirely determined by the difference in the physiological optimal trait values among the two species; (ii) with lower cost or higher pH snsitivity, the difference in the traits at evolutionary equilbrium is very small; and (iii) stronger interspecific competition (i.e., wider niche width) largerly increases the difference in the traits at evolutionary equilbrium.

The equation A1 can thus be transformed as:

$\frac{r_{1}X_{1}}{r_{2}X_{2}}=-\frac{A}{B}$, (A3)

where *A*= $c\left( \bar{p}_{2}-p_{2} \right)+\theta\left( Y-p_{2} \right)$ and *B*= $c\left( \bar{p}_{1}-p_{1} \right)+\theta\left( Y-p_{1} \right)$.

Without interspecific competition, the condition of evolutionary equilibrium becomes:

$c\left( \bar{p}_{2}-p_{2} \right)+\theta\left( Y-p_{2} \right)=c\left( \bar{p}_{1}-p_{1} \right)+\theta\left( Y-p_{1} \right)$ = 0. (A4)

Then, we have:

*p_i_*= $\frac{c\bar{p}_{i}+\theta Y}{c+\theta}$ . (A5)

which indicates that a magnitude relation of trait values at evolutionary equilbrium is determined by the physiological optimal trait value and pH, which are weighted by the relative effect of cost (*c*/(*c* + *θ*)) and pH sensitivity (*θ*/(*c* + *θ*)), respectively. When $\bar{p}_{1}<\bar{p}_{2}$; *p*_1_*^*^* *< p*_2_*^*^*, and vice versa.

Next, consider the ecological equilibrium when two competing species coexist (*dX_i_*/*dt* = 0). In this scenario, a ratio of equilibrium population sizes *X_i_^*^* is:

$\frac{X_{1}^{*}}{X_{2}^{*}}=\frac{{r_{01}D}_{2}-{r_{02}\alpha_{0}C}_{1}}{{r_{02}D}_{1}-{r_{01}\alpha_{0}C}_{2}}$, (A6)

where *C_i_* = $e^{c\left( \bar{p}_{i}-p_{i} \right)^{2}+\delta\left( p_{i}-p_{j} \right)^{2}+\theta\left( Y-p_{i} \right)^{2}}$ and *D_i_* = $e^{c\left( \bar{p}_{i}-p_{i} \right)^{2}+\theta\left( Y-p_{i} \right)^{2}}$. With an intermediate pH equilibrium (*k*_2_*X*_2_ – *k*_1_*X*_1_), the Eq. A6 becomes:

$\frac{k_{2}}{k_{1}}=\frac{{r_{01}D}_{2}-{r_{02}\alpha_{0}C}_{1}}{{r_{02}D}_{1}-{r_{01}\alpha_{0}C}_{2}}$, (A7)

Again, for analytical tractability, I consider a case without interspecific competition. Thus, at evolutionary equilibrium (Eq. A5) Eq. A7 becomes:

$\frac{k_{1}r_{01}}{k_{2}r_{02}}=e^{-\frac{c\theta\left( \bar{p}_{2}-\bar{p}_{1} \right)\left( \bar{p}_{2}+\bar{p}_{1}-2Y \right)}{c+\theta}}$ (A8)

Taking the logarithm of both sides, we obtain the intermediate pH equilibrium:

*Y^*^* = $\frac{\left( 1/c+1/\theta\right)\gamma}{2\left( \bar{p}_{2}-\bar{p}_{1} \right)}+\frac{\bar{p}_{1}+\bar{p}_{2}}{2}$, (A9)

where *γ =*log(*k*_1_*r*_01_/*k*_2_*r*_02_). At intermediate pH equilibrium, the population sizes of the bacteria species at the evolutionary equilibrium are given by:

*X_i_^*^ = r*_0_*_i_E_i_*, (A10)

where $E_{1}=e^{-\frac{\left[ \gamma\theta+c\left\{ \gamma+\theta\left( \bar{p}_{1}-\bar{p}_{2} \right)^{2} \right\} \right]^{2}}{4c\theta\left( c+\theta\right)\left( \bar{p}_{1}-\bar{p}_{2} \right)^{2}}}$and $E_{2}=e^{-\frac{\left[ \gamma\theta+c\left\{ \gamma-\theta\left( \bar{p}_{1}-\bar{p}_{2} \right)^{2} \right\} \right]^{2}}{4c\theta\left( c+\theta\right)\left( \bar{p}_{1}-\bar{p}_{2} \right)^{2}}}$. In this case, the ratio of population equilibrium is only determined by the pH change rate ratios among the bacteria species (*X*_1_*^*^*/*X*_2_*^*^= k*_2_/*k*_1_).

On the other hand, in the alkaliphilic and acidophilic equilibrium (*Y^*^ =*1 or –1), the population sizes of the bacteria species at evolutionary equilibrium are:

*X_i_^*^ = r*_0_*_i_F_i_*, (A11)

where $F_{i}=e^{-\frac{\left( 1-\bar{p}_{i} \right)^{2}}{\left( 1/c+1/\theta\right)}}$ in the alkaliphilic equilibrium and $F_{i}=e^{-\frac{\left( 1+\bar{p}_{i} \right)^{2}}{\left( 1/c+1/\theta\right)}}$ in the acidophilic equilibrium. In these cases, the ratio of population equilibrium becomes:

${\frac{X_{1}^{*}}{X_{2}^{*}}=\frac{r_{01}}{r_{02}}e}^{\frac{\left( \bar{p}_{2}-\bar{p}_{1} \right)\left( -{2+\bar{p}}_{2}+\bar{p}_{1} \right)}{\left( 1/c+1/\theta\right)}}$ (alkaliphilic equilibrium), (A12a)

${\frac{X_{1}^{*}}{X_{2}^{*}}=\frac{r_{01}}{r_{02}}e}^{\frac{\left( \bar{p}_{2}-\bar{p}_{1} \right)\left( {2+\bar{p}}_{2}+\bar{p}_{1} \right)}{\left( 1/c+1/\theta\right)}}$ (acidophilic equilibrium), (A12b)

which are determined not only by the ratio of maximum growth rates among the bacteria species but also by the difference between the physiologically optimal trait values.

The trait values in each pH equilibrium are given by:

*p*_L_*_i_^*^* = $\frac{c\bar{p}_{i}+\theta}{c+\theta}$ (alkaliphilic equilibrium), (A13a)

*p*_C_*_i_^*^* = $\frac{c\bar{p}_{i}-\theta}{c+\theta}$ (acidophilic equilibrium), (A13b)

*p*_I_*_i_^*^* = $\frac{c\bar{p}_{i}}{c+\theta}+\frac{\theta}{c+\theta}\cdot\frac{\left( \bar{p}_{1}{+\bar{p}}_{2} \right)}{2}+\frac{\gamma}{2c\left( \bar{p}_{2}{-\bar{p}}_{1} \right)}$ (intermediate equilibrium). (A13c)

The pH preferences in alkaliphilic and acidophilic equilibria (A13a, b) are determined not by the physiologically optimal trait value of the other species but by their own optimal trait values. When the cost effect (*c*) is smaller than the pH sensitivity effect (*θ*), a strong preference for an alkaline or acidic environment is likely to evolve as an alkaliphilic or acidophilic equilibrium, respectively. In intermediate equilibrium (A13c), the pH preferences of each species are determined by the physiologically optimal trait values of both species. With a parameter balance (*γ*= 0 or *k*_1_*r*_01_ = *k*_2_*r*_02_), we find that the preference is attracted to the optimal trait value of the other species, especially when the cost effect is smaller than the pH sensitivity effect. Thus, a strong preference for an alkaliphilic or acidophilic equilibrium is not likely to evolve. Without the parameter balance (*γ*$\neq$ 0), a strong preference for an alkaline or acidic environment can evolve, especially when the physiologically optimal trait values are similar.

Without interspecific competition, we can conduct a local stability analysis of the equilibrium in some simple situations. First, we focus on the alkaliphilic equilibrium (*Y^*^* = 1). For analytical tractability, we consider that the trait dynamics are very fast and at a quasi-equilibrium (Eq. A5). This situation implies that the trait changes are caused by faster adaptation compared to the population dynamics, such as phenotypic plasticity. In this situation, the system is defined by the following differential equations:

*dY*/*dt* = (*k*_2_*X*_2_ – *k*_1_*X*_1_)(1 – *Y*^2^), (A14a)

*dX_i_*/*dt* = (*r_i_*(*Y*) – *X_i_*)*X_i_*, (A14b)

The local stability of the system described by Eq. A14 is analyzed by linearizing the dynamics near the nontrivial equilibrium. We can judge the stability by whether the characteristic equation of the Jacobian matrix satisfies the Routh-Hurwitz criteria. Under the equilibrium condition (*Y* = 1, *X_i_ = r_i_*), we obtain the Jacobian matrix:

$J=\left( \begin{matrix} 2{(k}_{1}r_{1}-k_{2}r_{2}) & r_{1}{r_{1}}^{'} & r_{2}{r_{2}}^{'} \\ 0 & -r_{1} & 0 \\ 0 & 0 & -r_{2} \end{matrix} \right)$, (A15)

where the prime represents the derivation. The characteristic equation for determining the eigenvalues is λ^3^ + ω_1_λ^2^ + ω_2_λ + ω_3_ = 0, where ω_1_ = (*r*_1_ + *r*_2_) + 2(*k*_1_*r*_1_ + *k*_2_*r*_2_), ω_2_ = 2(*k*_2_*r*_2_^2^ – *k*_1_*r*_1_^2^) + *r*_1_*r*_2_(1 + 2*k*_2_ – 2*k*_1_), and ω_3_ = 2*r*_1_*r*_2_(*k*_2_*r*_2_ – *k*_1_*r*_1_). The equilibrium is locally stable if ω_1_, ω_3_ > 0 and ω_1_ω_2_ > ω_3_. Since the other two conditions are always met if ω_3_ > 0, the stability condition reduces to simply ω_3_ > 0:

*k*_2_/*k*_1_ > *r*_1_/*r*_2_ (A16)

By introducing the specific function of *r_i_*, we obtain the stability condition explicitly:

$\frac{k_{2}r_{02}}{k_{1}r_{01}}>e^{\frac{c\theta\left( \bar{p}_{2}-\bar{p}_{1} \right)\left( \bar{p}_{1}+\bar{p}_{2}-2 \right)}{c+\theta}}$ (A17)

Since *k*_2_*r*_02_/*k*_1_*r*_01_ = *e^−γ^*, Eq. A17 becomes:

*γ*(1/*c* + 1/*θ*) < $\left( \bar{p}_{2}-\bar{p}_{1} \right)\left( {2-\bar{p}}_{1}-\bar{p}_{2} \right)$ (A18)

Given an appropriate parameter range of the physiological optimal trait values,$-1<\bar{p}_{i}<1$, we obtain the condition of $\bar{p}_{2}$ for stability:

$\bar{p}_{2}>\hat{p}_{2}=1-\sqrt{\left( 1-\bar{p}_{1} \right)^{2}-\gamma\left( \frac{1}{c}+\frac{1}{\theta} \right)}$, (A19)

where $\hat{p}_{2}$ is the threshold of $\bar{p}_{2}$ for stability. This is the same when the condition under which the intermediate pH equilibrium (A9) is smaller than 1. When *γ <*0, then $\hat{p}_{2}{<\bar{p}}_{1}$. When *γ >*0, then $\hat{p}_{2}{>\bar{p}}_{1}$. The trait values at a critical value, $\hat{p}_{2}$, are:

*p*_1_ = $\frac{c\bar{p}_{1}+\theta}{c+\theta}$ (A20a)

*p*_2_ = $\frac{c\hat{p}_{2}+\theta}{c+\theta}$ (A20b)

Consider a simple case where *γ*= 0 (*k*_1_*r*_01_ = *k*_2_*r*_02_). The stability condition (A19) is reduced to:

$\bar{p}_{1}<\bar{p}_{2}$ (A21)

Similarly, at acidophilic equilibrium (*Y^*^* = –1), we obtain the stability condition:

*γ*(1/*c* + 1/*θ*) > $\left( \bar{p}_{1}-\bar{p}_{2} \right)\left( {2+\bar{p}}_{1}+\bar{p}_{2} \right)$ (A22)

The condition of $\bar{p}_{2}$ for stability is:

$\bar{p}_{2}>\check{p}_{2}=-1+\sqrt{\left( 1+\bar{p}_{1} \right)^{2}-\gamma\left( \frac{1}{c}+\frac{1}{\theta} \right)}$, (A23)

where $\check{p}_{2}$ is the threshold of $\bar{p}_{2}$ for stability. This is the same when the condition under which the intermediate pH equilibrium (A9) is larger than –1. When *γ >*0, then $\check{p}_{2} {<\bar{p}}_{1}$. When *γ <*0, then $\check{p}_{2}{>\bar{p}}_{1}$. Thus, the trait values at a critical value, $\check{p}_{2}$, are:

*p*_1_ = $\frac{c\bar{p}_{1}-\theta}{c+\theta}$ (A24a)

*p*_2_ = $\frac{c\hat{p}_{2}-\theta}{c+\theta}$ (A24b)

The stability condition (A23) in a simple case where *γ*= 0 is: $\bar{p}_{1}<\bar{p}_{2}$, which is the same as that of alkaliphilic equilibrium (Eq. A21). Notice that when *γ*= 0, $\hat{p}_{2}$=$\check{p}_{2}$=$\bar{p}_{1}$.

Finally, we will analyze the local stability of the intermediate pH equilibrium. Under the equilibrium condition (*k*_2_*X*_2_ – *k*_1_*X*_1_, *X_i_ = r_i_*), we obtain the Jacobian matrix:

$J=\left( \begin{matrix} 0 & r_{1}{r_{1}}^{'} & r_{2}{r_{2}}^{'} \\ k_{1}(Y^{2}-1) & -r_{1} & 0 \\ k_{2}({1-Y}^{2}) & 0 & -r_{2} \end{matrix} \right)$ (A25)

The characteristic equation for determining the eigenvalues is λ^3^ + ω_1_λ^2^ + ω_2_λ + ω_3_ = 0, where ω_1_ = *r*_1_ + *r*_2_; ω_2_ = *r*_1_*r*_2_ – *k*_2_*r*’_2_(1 – *Y*^*2^) + *k*_1_*r*’_1_(1 – *Y*^*2^); and ω_3_ = (*Y*^*2^ – 1)*r*_1_*r*_2_(*k*_1_*r*’_1_ – *k*_2_*r*’_2_). The equilibrium is locally stable if ω_1_, ω_3_ > 0 and ω_1_ω_2_ > ω_3_. The first condition (ω_1_ > 0) is always held. Given an appropriate range – 1 < *Y*< 1, the condition, ω_3_ > 0, is met if

*k*_1_*r*’_1_ < *k*_2_*r*’_2_. (A26)

By substituting the intermediate equilibrium, Eq. A26 becomes:

$\bar{p}_{1}>\bar{p}_{2}$ (A27)

The last condition, ω_1_ω_2_ > ω_3_, is met if:

*r*_1_ + *r*_2_ + (*Y*^*2^ – 1) {(*r*_2_/*r*_1_)*k*_2_*r*’_2_ – (*r*_1_/*r*_2_)*k*_1_*r*’_1_} > 0 (A28)

A sufficient condition for Eq. A28 is:

(*r*_2_/*r*_1_)*k*_2_*r*’_2_ < (*r*_1_/*r*_2_)*k*_1_*r*’_1_ (A29)

By substituting the intermediate equilibrium and using a necessary stability condition (A27), Eq. A29 becomes:

$\bar{p}_{2}>\bar{p}_{1}-\sqrt{\left( 1-\frac{2}{1+\left( \frac{k_{1}}{k_{2}} \right)^{2}} \right)\gamma\left( \frac{1}{c}+\frac{1}{\theta} \right)}$ (A30)

This requires: *k*_2_ > *k*_1_ when *γ* < 0 and *k*_2_ < *k*_1_ when *γ*> 0. When *γ*= 0, this is the same with the necessary condition for stability (A27).

Next, we examine the evolutionary equilibrium of trait values based on the local stability condition. At alkaliphilic and acidophilic equilibrium (A13a, b), *p*_2_^*^ > *p*_1_^*^ is always held because of the stability condition ($\bar{p}_{1}<\bar{p}_{2}$). This implies that each type of bacteria (alkaline producing and acid producing) can evolve to prefer the pH made by its self-produced products (*p*_2_^*^ > 0 > *p*_1_^*^), but, the opposite case (*p*_2_^*^ < 0 < *p*_1_^*^) is impossible. That is, both bacteria types cannot evolve to prefer the pH environment made by the other bacteria. In addition, the trait values at an alkaliphilic equilibrium are larger than those found at an acidophilic equilibrium (i.e., *p*_L_*_i_*^*^ > *p*_C_*_i_*^*^). The difference in trait values of each bistable equilibrium is 2*θ*/(*c* + *θ*). In both equilibria, *p*_2_^*^ > *p*_1_^*^ > 0 and 0 > *p*_2_^*^ > *p*_1_^*^ are possible, implying that both species can evolve to prefer either an alkaline or acidic environment. At intermediate equilibrium (A13c), *p*_2_^*^ < *p*_1_^*^ is always held because of the stability condition ($\bar{p}_{2}<\bar{p}_{1}$). This implies that each type of bacteria (alkaliphilic and acidophilic) can evolve to prefer the pH made by the other bacteria (*p*_2_^*^ < 0 < *p*_1_^*^). The opposite case (*p*_2_^*^ > 0 > *p*_1_^*^) is impossible. Both bacteria types cannot evolve to prefer the pH made by their self-produced products. This is a contrast between alkaliphilic or acidophilic equilibriums.

In a similar way, consider a simple case with only acidophilic species. Assuming a quasi-equilibrium of the trait dynamics, we obtain the Jacobian matrix at the acidophilic equilibrium (*Y* = –1):

$J=\left( \begin{matrix} -2br_{1} & r_{1}{r_{1}}^{'} \\ 0 & -r_{1} \end{matrix} \right)$ (A31)

The characteristic equation for determining the eigenvalues is λ^2^ + ω_1_λ + ω_2_ = 0, where ω_1_ = *r*_1_ + 2*r*_1_*k*_1_ and ω_2_ = 2*k*_1_*r*_1_^2^. The equilibrium is locally stable because the stability condition, ω_1_, ω_2_ > 0, is always held. Under alkaliphilic equilibrium (*Y* = 1), the Jacobian matrix is:

$J=\left( \begin{matrix} 2br_{1} & r_{1}{r_{1}}^{'} \\ 0 & -r_{1} \end{matrix} \right)$ (A32)

The coefficients of the characteristic equation are ω_1_ = *r*_1_ – 2*r*_1_*k*_1_ and ω_2_ = –2*k*_1_*r*_1_^2^. The equilibrium is locally unstable because ω_2_ < 0 is always held. This analysis applies to a case with only alkaliphilic species. The result is completely opposite: alkaliphilic equilibrium is stable but acidophilic equilibrium is unstable.

Consider a case where both species change their pH in the same direction and assume that both species produce an alkaline substance. In this example, *k*_1_ < 0 in Eq. (1). Notice that there is no intermediate pH equilibrium. In addition, the assumption, *k*_1_ < 0, meets the stability criteria of the alkaliphilic equilibrium (Eq. A15), but does not meet the criteria in the acidophilic equilibrium (Eq. A25). This implies that only the alkaliphilic equilibrium is stable. If the same logic is applied when both species produce an acidic substance, then *k*_2_ < 0 and only the acidophilic equilibrium is stable. In the absence of evolutionary change, an acidophilic species has an equilibrium state: *X*_1_*^*^ = r*_01_$e^{-\theta\left( 1-\bar{p}_{1} \right)^{2}}$. When acidophilic species prefer an alkaline environment, they can go toward extinction, especially when they have a high pH sensitivity. In such cases, adaptive pH changes rescue both species.

A lack of evolutionary adaptation is approximately the same as when the cost constraint (*c*) is very large. In this case, the thresholds separating the regimes can be different from the system under adaptation. When *γ* = 0, the threshold between the systems does not change with or without adaptation, because it does not include *c*. When *γ*$\neq$ 0, it does change, because it includes *c* (Eqs. A19, A23). Without adaptation, in the thresholds, 1/*c* = 0. Hence, a large value of *γ*/*c* largely changes the thresholds predicted from ecological theory.

# Supplemental Figures

**Figure S1.** An example of the consequences of eco-evolutionary dynamics without interspecific competition. (a) pH dynamics. In (b, c) and (d, e), the equilibrium points of population size and trait values are illustrated, respectively. In a bistable regime ($\bar{p}_{2}>\bar{p}_{1}$), the system has different equilibria. (b, d) Alkaliphilic equilibrium. (c, e) Acidophilic equilibrium. In this case, *p*_1_^*^ *<*0 < *p*_1_^*^ is possible in a range of $\bar{p}_{2}<\bar{p}_{1}$. Parameters are the same with those of Fig. 1a except for$\bar{p}_{1}$ *=*0.1. The other information is the same as Figs. 1a and 2.

**Figure S2.** Resilience of equilibrium in a case with low cost. Resilience is illustrated in two cases: (a) Alkaliphilic equilibrium; and (b) Acidophilic equilibrium. Note that in the areas where $\bar{p}_{2}<\bar{p}_{1}$, the two cases have the same resilience (of the intermediate equilibrium). The information and parameters are the same as those of Fig. 3, except for *c ­=*1.


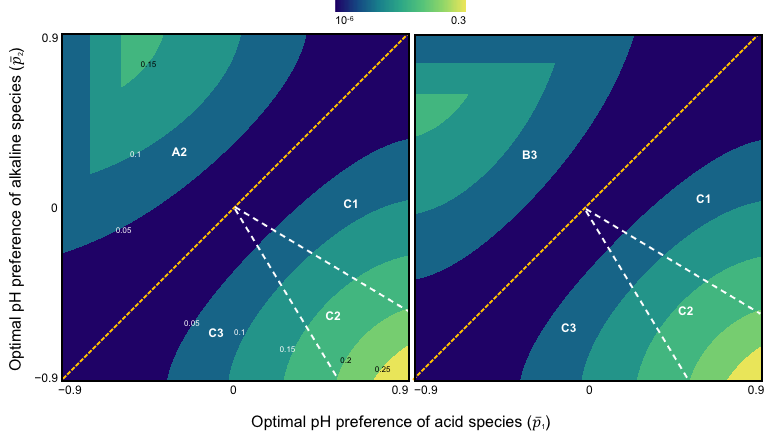


**Figure S3.** Resilience of equilibrium in a case with high cost. Resilience is illustrated in two cases: (a) Alkaliphilic equilibrium; and (b) Acidophilic equilibrium. Note that in the areas where $\bar{p}_{2}<\bar{p}_{1}$, the two cases have the same resilience (of the intermediate equilibrium). The information and parameters are the same as those of Fig. 3, except for *c ­=* 10.


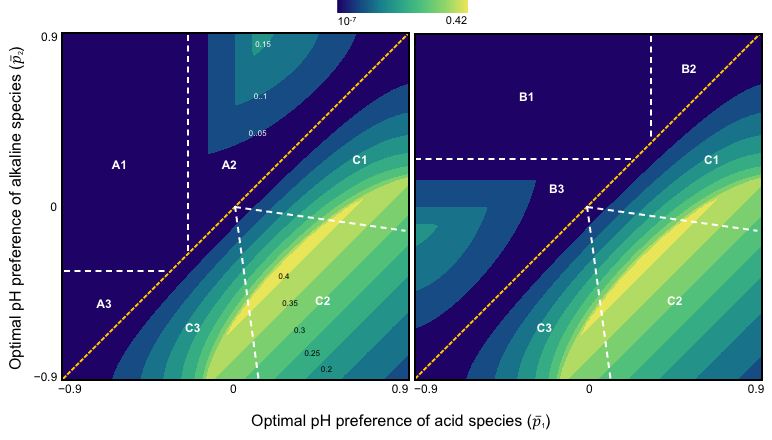


**Figure S4.** Resilience of equilibrium in a case with low pH sensitivity. Resilience is illustrated in two cases: (a) Alkaliphilic equilibrium; and (b) Acidophilic equilibrium. Note that in the areas where $\bar{p}_{2}<\bar{p}_{1}$, the two cases have the same resilience (of the intermediate equilibrium). The information and parameters are the same as those of Fig. 3, except for *θ ­=* 1.


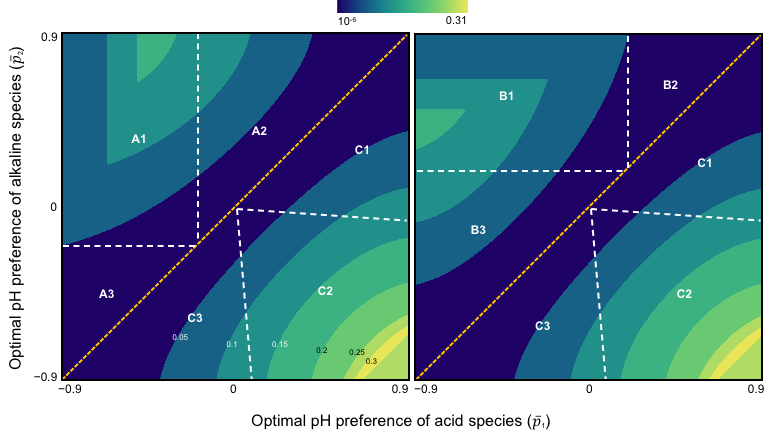


**Figure S5.** Resilience of equilibrium in a case with high pH sensitivity. Resilience is illustrated in two cases: (a) Alkaliphilic equilibrium; and (b) Acidophilic equilibrium. Note that in the areas where $\bar{p}_{2}<\bar{p}_{1}$, the two cases have the same resilience (of the intermediate equilibrium). The information and parameters are the same as those of Fig. 3, except for *θ ­=* 5.


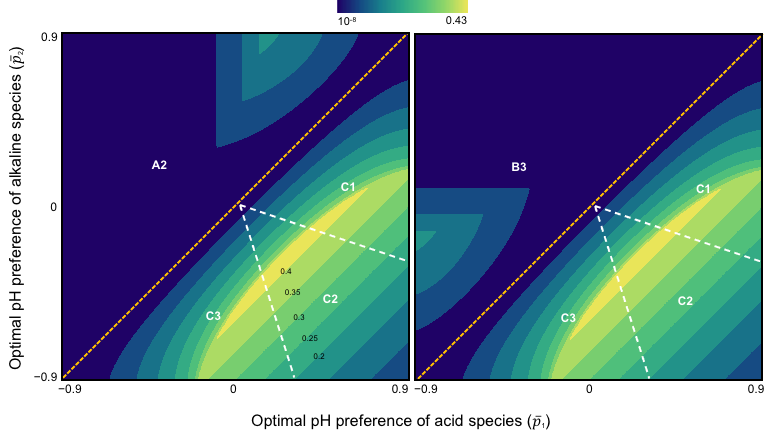


**Figure S6.** Effects of parameter imbalance on the consequences of eco-evolutionary dynamics without interspecific competition. (a) pH dynamics. In (b, c) and (d, e), the equilibrium points of population size and trait values are illustrated, respectively. In a bistable regime ($\bar{p}_{2}>\check{p}_{2}$), the system has different equilibria. Within a range, ${\hat{p}_{2}<\bar{p}}_{2}<\check{p}_{2}$, alkaliphilic equilibrium is a uniquely stable equilibrium. See SI text for details of $\hat{p}_{2}$ and $\check{p}_{2}$. (b, d) Alkaliphilic equilibrium. (c, e) Acidophilic equilibrium. The parameters are the same as those of Fig. 1a except for *k*_2_ = 0.2. The other information is the same as Figs. 1a and 2.

**Figure S7.** Resilience of equilibrium in a case with parameter imbalance of *r*_0_*_i_*. Resilience is illustrated in two cases: (a) Alkaliphilic equilibrium; and (b) Acidophilic equilibrium. The information and parameters are the same as those of Fig. 3c, d, except for *r*_02_ = 2.


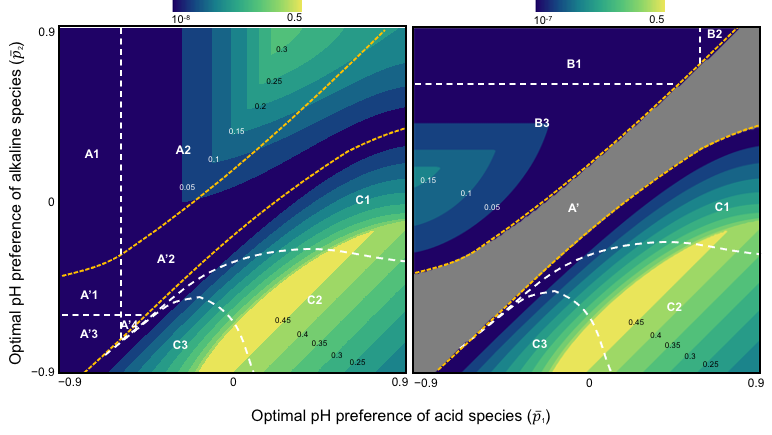


**Figure S8.** Predictions on ecological consequences from ecological theory and eco-evolutionary theory. (a) Changes in a parameter region of three regimes due to evolutionary effect. Dashed lines indicate the thresholds separating regimes A, A’ and C, as shown in Fig. 3c. The parameters are the same as those of Fig. 3c in yellow lines, green lines (except for *c =* 2) and black lines (except for *c =* ∞). Note that black lines are based on ecological theory without evolution. Each parameter space with different combinations of symbols shows either same regime or different regime among ecological theory and eco-evolutionary theory with different values of cost (different colors in symbols correspond to each line with the same color). (b) Predictions from ecological theory and eco-evolutionary theory based on a situation where evolutionary stable trait values are fixed to the physiologically optimal values. Parameters are the same as those of Fig. 3c except for *θ* = 2. Dashed black and solid yellow lines are the thresholds separating different regimes in ecological theory and eco-evolutionary theory, respectively. Open and closed stars represent the predictions from ecological theory and eco-evolutionary theory, respectively. The stars with same color have a same trait values at the equilibrium. In black, red and blue open stars, ($\bar{p}_{1},\bar{p}_{2})=$ (–0.1, 0.1), (–0.2, 0.1) and (–0.2, 0), respectively. In black, red and blue closed stars, ($\bar{p}_{1},\bar{p}_{2})=$ (–0.54, –0.26), (–0.68, –0.26) and (–0.68, –0.4), respectively, each of which makes the trait values at the equilibrium same with optimal values of the open stars with same color. In these examples, ecological theory (open stars) predicts bi-stability, while eco-evolutionary theory (closed stars) predicts mono-stability with an extreme pH, in spite of same equilibrium trait values.

**Figure S9.** An example of the consequences of eco-evolutionary dynamics with weak interspecific competition. (a) pH dynamics. In (b, c) and (d, e), the equilibrium points of population size and trait values are illustrated, respectively. In a bistable regime, the system has different equilibria. (b, d) Alkaliphilic equilibrium. (c, e) Acidophilic equilibrium. The parameters are the same as those of Fig. 1b.


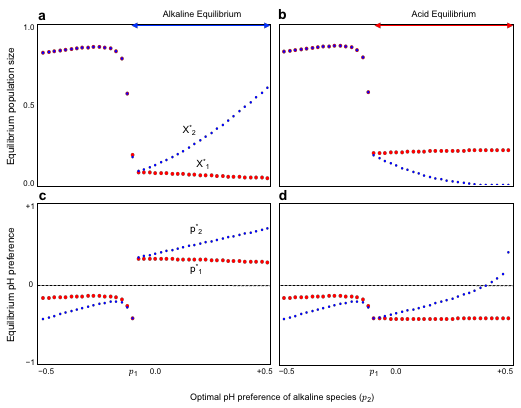


**Figure S10.** Eco-evolutionary consequences in cases with interspecific competition. (a) Weak interspecific competition. (b) Strong interspecific competition. In A, the equilibria are bistable. In the figures, only the alkaliphilic equilibrium is shown. In B’ (gray region), the alkaliphilic species goes extinct (*X*_2_ = 0). In C, the system goes to a unique equilibrium. In the blue regions, the acidophilic species goes extinct. In the green regions, the two species coexist. The numbers in A and C represent the same as those of Fig. 3. A direct simulation was used to calculate the equilibrium states of each variable, with the mean values of each variable calculated using the last 5000 time steps of each variable during 20000 steps. The extinction threshold was assumed to be 10^-5^ (if a population size is less than this value, the species is assumed to be extinct). The parameters are the same as those of Fig. 1b and 1c in (a) and (b), respectively. The initial values are: *X_i_*(0) = 0.1; *Y*(0) = 0.9; and *p_i_*(0) = $\bar{p}_{i}$.


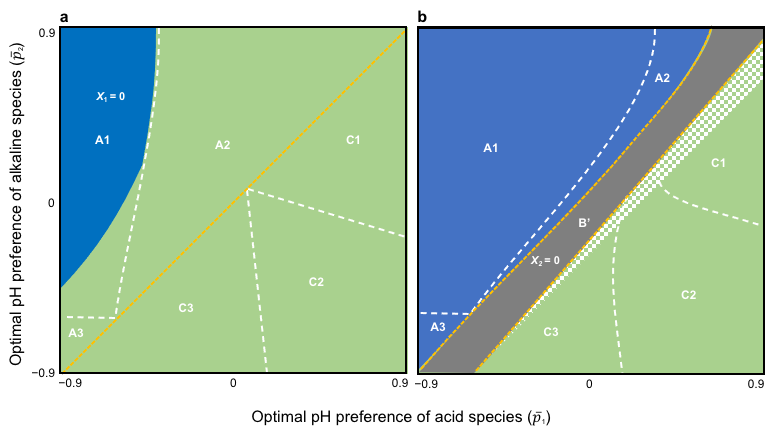


**Figure S11.** An example of the consequences of eco-evolutionary dynamics with strong interspecific competition. In (a, b) and (c, d), the equilibrium points of population size and trait values are illustrated, respectively. In a bistable regime, the system has different equilibria. (a, c) Alkaliphilic equilibrium. (b, d) Acidophilic equilibrium. The red and bule lines represent the maximum and minimum values of population and trait oscillation, respectively. In the gray regions, one species is competitively excluded. The parameters are the same as those of Fig. 1c.


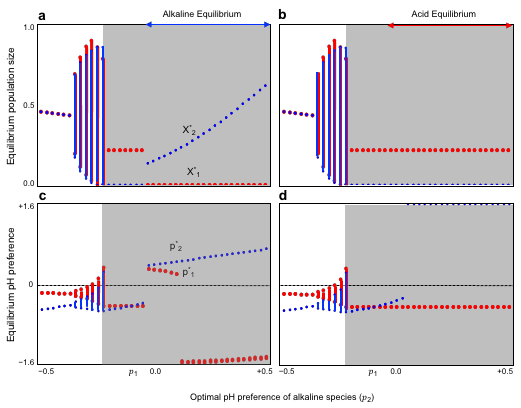


**Figure S12.** An example of the consequences of eco-evolutionary dynamics with strong interspecific competition. (a) pH dynamics. In (b, c) and (d, e), the equilibrium points of population size and trait values are illustrated, respectively. In a bistable regime, the system has different equilibria. (b, d) Alkaliphilic equilibrium. (c, e) Acidophilic equilibrium. The red and bule lines represent the maximum and minimum values of population and trait oscillation, respectively. In the gray regions, one species is competitively excluded. The parameters are the same as those of Fig. 1c, except for $\bar{p}_{1}$*=*0.1.


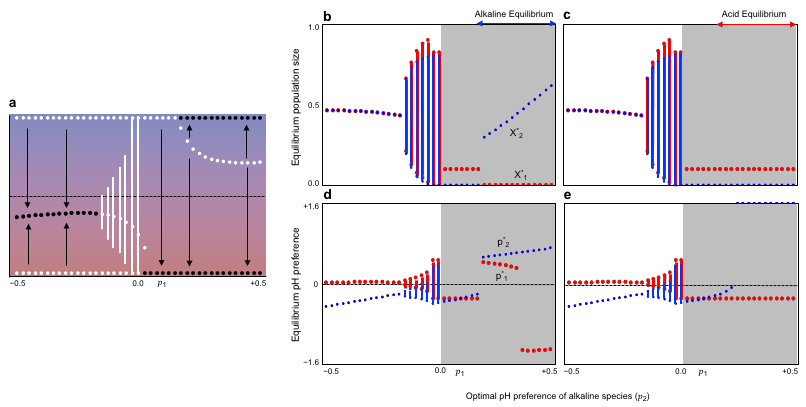


**Figure S13.** Effects of adaptation speed on dynamics. (a) *G_i_* = 0.01; (b) *G_i_* = 0.02; (c) *G_i_* = 0.1; (d) *G*_1_ = 0.1; *G*_2_ = 0.01; (e) *G*_1_ = 0.01; *G*_2_ = 0.1. The parameters and initial values are the same as those of Fig. 1c, except for $\bar{p}_{2}$*=*–0.3. Note that the time range is the last 2000 steps during 20000 steps.


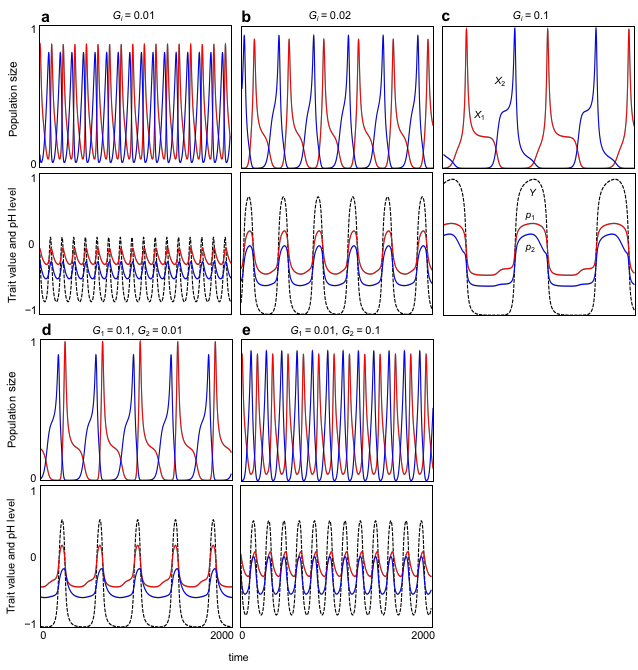


**Figure S14.** Parameter space of a limit cycle. The figures illustrate the effects of the parameters (*c*, *θ*, and *δ*) on the stability of the equilibrium. In the cycle regions, limit cycles occur. In the equilibrium regions, the system approaches the equilibrium. The other parameters are the same as those of Fig. 1c.


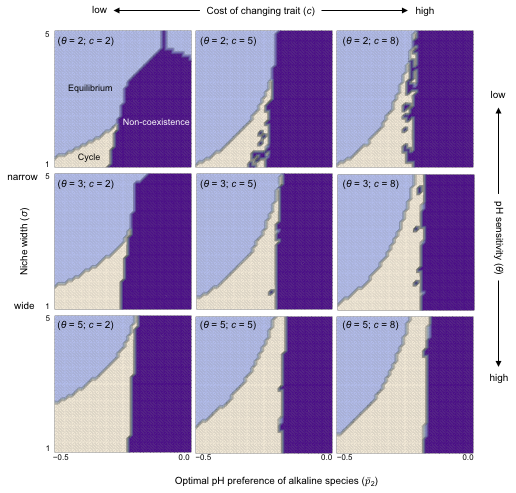


**Figure S15.** Effects of adaptation speed on the stability of oscillations. Contours represent the coefficient of variation (CV), which indicates the stability of the population dynamics. When CV is low, the stability is high and coexistence would likely be maintained. The last 5000 time steps of the population sizes of each species was used to calculate CV. The parameters, initial conditions and time steps are the same as the those of Fig. S13.


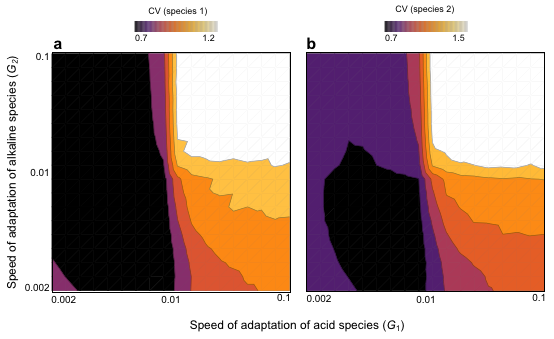

Supplement: Supplementary file 1 — Supplementary Information. [file 41598_2023_36221_MOESM1_ESM.docx]
